# Pattern formation in multiplex networks

**DOI:** 10.1038/srep10840

**Published:** 2015-06-04

**Authors:** Nikos E. Kouvaris, Shigefumi Hata, Albert Díaz- Guilera

**Affiliations:** 1 Department of Physics, University of Barcelona, Martí i Franqués 1, E-08028, Barcelona, Spain; 2 Department of Mathematical Science and Advanced Technology, Japan Agency for Marine-Earth Science and Technology, 236-0001 Kanagawa, Japan

## Abstract

The advances in understanding complex networks have generated increasing interest in dynamical processes occurring on them. Pattern formation in activator-inhibitor systems has been studied in networks, revealing differences from the classical continuous media. Here we study pattern formation in a new framework, namely multiplex networks. These are systems where activator and inhibitor species occupy separate nodes in different layers. Species react across layers but diffuse only within their own layer of distinct network topology. This multiplicity generates heterogeneous patterns with significant differences from those observed in single-layer networks. Remarkably, diffusion-induced instability can occur even if the two species have the same mobility rates; condition which can never destabilize single-layer networks. The instability condition is revealed using perturbation theory and expressed by a combination of degrees in the different layers. Our theory demonstrates that the existence of such topology-driven instabilities is generic in multiplex networks, providing a new mechanism of pattern formation.

Distributed active media support a variety of self-organized patterns, such as stationary and oscillatory structures, spiral waves, and turbulence^1^,^2^,^3^. Such media are often described by reaction-diffusion systems and consist of elements obeying an activator-inhibitor dynamics with local coupling. In his pioneering paper^1^, Turing showed that a uniform steady state can be spontaneously destabilized, leading to a spontaneous formation of a periodic spatial pattern, when reacting species diffuse with different mobilities. It was later proposed by Gierer and Meinhardt^4^ that an activator-inhibitor chemical reaction is a typical example achieving Turing’s scenario. Turing instability is a classical mechanism of self-organization far from equilibrium, and plays an important role in biological morphogenesis. It has been extensively studied in biological^4^,^5^,^6^ and chemical^7^ systems, as well as real ecosystems^8^,^9^.

The active elements can also be coupled in more complicated ways, forming complex networks^10^,^11^. Complex networks are ubiquitous in nature^12^; two typical examples are epidemics spreading over transportation systems^13^ and ecological systems where distinct habitats communicate through dispersal connections^14^,^15^,^16^,^17^. Theoretical studies of reaction-diffusion processes on complex networks have recently attracted much attention^12^,^18^,^19^,^20^,^21^. Othmer and Scriven^22^,^23^ developed the general mathematical framework to describe Turing instability in networks, and provided several examples of small regular lattices. Afterwards, Turing patterns were explored in small networks of chemical reactors^24^,^25^. More recent work in this area includes detailed studies of Turing bifurcation and related hysteresis phenomena in large complex networks^26^,^27^, and oscillatory Turing patterns in multi-species ecological networks^28^.

In nature, the active elements of a system can communicate through different types of pathways with different architecture. Such a system with multiple types of links can be represented as a special type of complex network called a *multiplex network*^29^. Recent theoretical studies have shown that the spectral properties of multiplex networks are significantly different from those of single-layer networks^29^,^30^,^31^,^32^,^33^, and that these differences affect the diffusion processes occurring on the network^30^,^31^. Consequently, the emergent dynamics can exhibit new kinds of patterns. Examples include the breathing synchronization of cross-connected phase oscillators^34^ and the emergence of a metacritical point in epidemic networks, where diffusion of awareness is able to prevent infection and control the spreading of a disease^35^. Moreover, Asllani *et al.* studied Turing patterns in the context of multiplex networks^36^, where it was found that an additional inter-layer diffusion process can induce instabilities even if they are prevented in the isolated layers.

It has been reported that many man-made networks and real ecosystems are spatially fragmented in such a way that different species can migrate using different paths in separate layers^37^,^38^,^39^,^40^,^41^. In studies of classical swine fever, for example, it was found that an individual can spread the infection by different types of contacts characterized by different infection rates^37^. Moreover, the role of different but overlapping transportation networks was considered in a study exploring the diffusion pattern of severe acute respiratory syndrome near Beijing^38^.

This literature leads us to consider a new class of dynamical systems, *multiplex reaction networks*, where reacting species are transported over their own networks in distinct layers, but can react with each other across the inter-layer connections. This paper provides a general framework for multiplex reaction networks and constructs a theory for self-organized pattern formation in such networks. As a typical example, we investigate a diffusively-coupled activator-inhibitor system where Turing patterns can develop.

## Multiplex reaction networks

**Figure 1:**
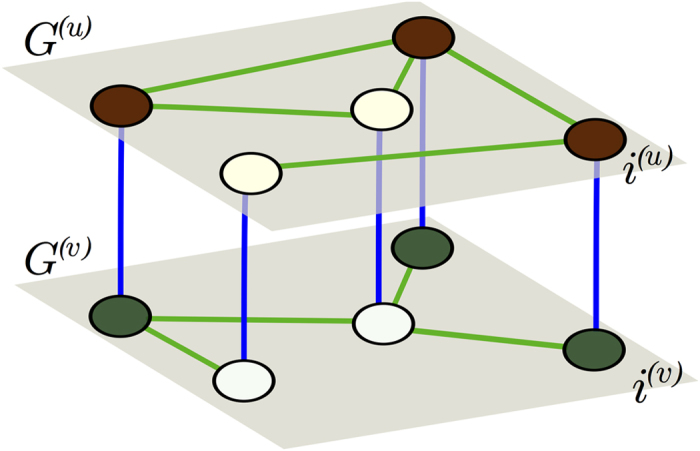
Activator-inhibitor system organized in multiplex network. Activator and inhibitor species occupy nodes in separate layers *G*^(*u*)^ and *G*^(*v*)^, respectively. They react across the layers (blue inter-layer links), while they migrate within their own layers (green intra-layer links).

We consider multiplex networks of activator and inhibitor populations, where the different species occupy separate network nodes in distinct layers. Species react across layers according to the mechanism defined by the activator-inhibitor dynamics, and diffuse to other nodes in their own layer through connecting links (see Fig. 1). Such a process can be described by the equations


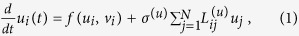



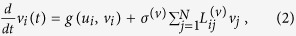


where *u*_*i*_ and *v*_*i*_ are the densities of activator and inhibitor species in nodes *i*^(*u*)^ and *i*^(*v*)^ of layers *G*^(*u*)^ and *G*^(*v*)^, respectively. The superscripts (*u*) and (*v*) refer to activator and inhibitor. The activator nodes are labeled by indices *i* = 1, 2…,*N* in order of decreasing connectivity. The same index ordering is applied to the inhibitor layer. The functions *f*(*u*_*i*_,*v*_*i*_) and *g*(*u*_*i*_,*v*_*i*_) specify the activator-inhibitor dynamics. The Laplacian matrices *L*^(*u*)^ and *L*^(*v*)^ describe diffusion processes in the two layers, and the constants *σ*^(*u*)^ and *σ*^(*v*)^ are the corresponding mobility rates (see details in the Methods section).

As a particular example we consider the Mimura-Murray ecological model^42^ on a multiplex network consisting of two scale-free layers. In the absence of diffusive coupling, such that *σ*^(*u*)^ = 0 and *σ*^(*v*)^ = 0, the multiplex system relaxes to a uniform state, *i.e.* (*u*_*i*_,*v*_*i*_) = (*u*_0_,*v*_0_) for all *i* = 1,…,*N*. The homogeneous densities are determined by *f* (*u*_0_,*v*_0_) = *g*(*u*_0_,*v*_0_) = 0 (see Methods). Under certain conditions, which we present here, Turing-like patterns can evolve from an instability driven by the multiplex structure.

## Linear stability of the uniform state

In simplex networks, where *L*^(*u*) ≡ ^*L*^(*v*)^, the uniform state may undergo a Turing instability as the ratio *σ*^(*v*)^/*σ*^(*u*)^ increases and exceeds a certain threshold. The instability leads to the spontaneous emergence of stationary patterns consisting of nodes with high or low densities of activators^26^. Such diffusion-induced instability can also take place in multiplex reaction networks (1)-(2). This phenomenon can be explained through a linear stability analysis with non-uniform perturbations. We introduce small perturbations, 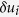 and 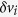, to the uniform steady state, as follows: (*u*_*i*_,*v*_*i*_) = (*u*_0_,*v*_0_) + (*δ*_*ui*_, *δ*_*vi*_). We then substitute the perturbed state into equations (1)-(2), 2 to obtain a set of coupled linearized differential equations. Finally, by means of an approximation technique described fully in the Methods section, we obtain a characteristic equation for the growth rate *λ* of the perturbations for each pair of nodes.

The onset of the instability occurs when Re *λ* = 0 for some pair of nodes *i*^(*u*)^ and *i*^(*v*)^. The instability condition is fulfilled when these nodes possess a combination of degrees *k*^(*u*)^ and *k*^(*v*)^ such that, the equation


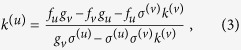


is satisfied. Here, *f*_*u*_, *f*_*v*_, *g*_*u*_ and *g*_*v*_ are partial derivatives at the uniform steady state. Condition (3) implies that a sufficiently large value of *σ*^(*v*)^ brings about instability, in the same manner as the Turing instability. However, an alternative scenario of the instability is revealed by equation (3). This can happen by increasing *k*^(*v*)^, even if the mobilities are equal (*σ*^(*u*)^ = *σ*^(*v*)^). This instability occurs in a strikingly different regime from classical Turing instabilities.

**Figure 2:**
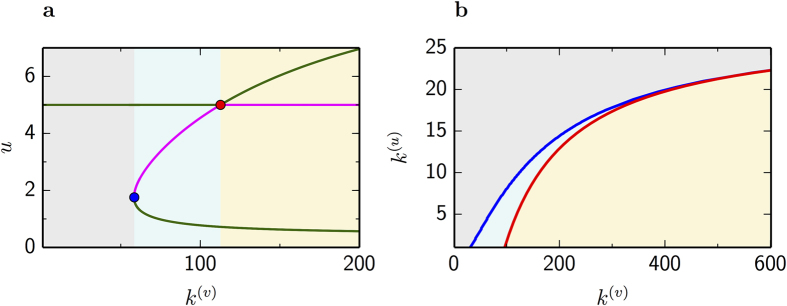
Bifurcation diagram. (**a)**, Stationary solutions of system (1)-(2) for *k*^(*u*)^ = 4. Green curves indicate stable solutions while magenta curves correspond to unstable solutions of the linearized system. Red point indicates the transcritical bifurcation where the uniform steady state (*u*_0_,*v*_0_) = (5,10) becomes unstable. Blue point corresponds to a saddle-node bifurcation of a solution (*u*,*v*) which originates from the transcritical bifurcation. (**b)**, Transcritical bifurcation (red curve) given by equation (3), is shown together with the continuation of the saddle-node bifurcation (blue curve) in the plane *k*^(*v*)^-*k*^(*u*)^.

Figure 2a shows the linear stability of system (1)-(2) for varying *k*^(*v*)^, holding *k*^(*u*)^ fixed. We clearly see that the uniform steady state is always a solution of the multiplex system. It is linearly stable (green line) for small values of *k*^(*v*)^. But at some critical value of *k*^(*v*)^ which satisfies equation (3), the system undergoes a transcritical bifurcation (red point) and becomes unstable (magenta line). Two new branches of solutions arise from the transcritical bifurcation. The unstable branch (magenta line) undergoes a second bifurcation (blue point), this time a saddle-node, giving rise to a new branch of stable solutions (green line) different from the uniform steady state. Figure 2b shows the transcritical (red line) and the saddle-node bifurcation (blue line) in the *k*^(*v*)^-*k*^(*u*)^ plane. The curve of the transcritical bifurcation is given by equation (3), while the curve of the saddle-node bifurcation has been derived by numerical continuation. One can see from equation (3) that by increasing *k*^(*v*)^, the boundary curve (red line) asymptotically approaches *k*^(*u*)^ = *f*_*u*_/*σ*^(*u*)^. This indicates that the instability can be observed if a node has sufficiently large *k*^(*v*)^, while its cunterpart has degree *k*^(*u*)^ less than the value mentioned above. This fact reveals an important difference from the classical Turing instability, which always takes place by increasing *σ*^(*v*)^ irrespective of *σ*^(*u*)^^26^.

The diffusion-induced instability occurs on the transcritical bifurcation. However, Turing patterns can also develop after the saddle-node bifurcation. In other words, we find that multiplex systems exhibit multistability in the area between these two bifurcations (cyan), where a branch of stable solutions coexists with the uniform steady state.

## Pattern formation arising from the instability

Suppose that the multiplex system starts almost in the uniform steady state with small perturbations. Equation (3) allows us to identify pairs of nodes (*i*^(*v*)^, *i*^(*u*)^) where the small perturbations will be amplified, so that these nodes leave the uniform state, triggering the formation of a non-uniform stationary pattern. Such a pattern cannot develop from pairs of nodes possessing degrees in the grey area of Fig. 2b, where only the uniform state exists. However, pairs of nodes with degrees in the yellow area, beyond the transcritical bifurcation, are unstable. Under small perturbations they can leave the uniform state, yielding the formation of a stationary non-uniform pattern. The cyan area between the two bifurcations indicates that the system exhibits multistability, where the uniform steady state coexists with a branch of solutions corresponding to non-uniform patterns.

**Figure 3:**
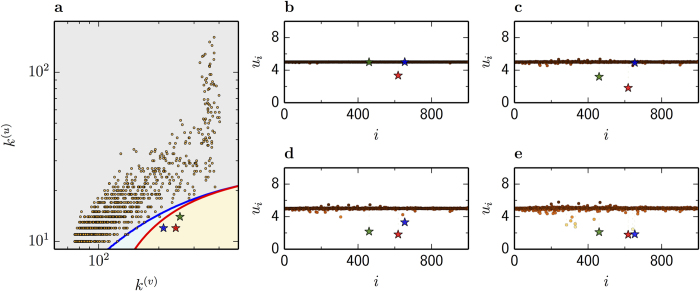
Multiplex diffusion-induced instability. (**a)**, Degree combination for pairs of nodes *i*^(*v*)^ and *i*^(*u*)^ is shown in plane *k*^(*v*)^-*k*^(*u*)^ together with the curves of saddle-node (blue) and transcritical (red) bifurcations. Snapshots of the activator pattern for *t* = 50 (**b**), *t* = 63 (**c**), *t* = 70 (**d**) and the fully developed pattern for *t* = 500 (**e**) are shown for the Mimura-Murray model with *σ*^(*v*)^ = *σ*^(*u*)^ = 0.12 on a multiplex network with scale-free layers of *N* = 1,000 nodes and mean degrees 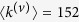 and 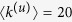 (see also Supplementary Movie S1). Nodes are ordered according to decreasing degrees *k*^(*u*)^.

We verify this scenario for a multiplex network where both layers, *G*^(*v*)^ and *G*^(*u*)^, are scale-free. Figure 3a displays the actual degree combination (*k*^(*v*)^, *k*^(*u*)^) for each pair of nodes *i*^(*v*)^, *i*^(*u*)^ (orange points) of this network in the *k*^(*v*)^-*k*^(*u*)^ plane, together with the bifurcation curves. Three pairs of nodes, the critical ones, denoted by stars, have degrees exceeding the instability threshold. Thus, a non-uniform pattern starts to grow from these nodes. The critical node denoted by the red star is the first to spontaneously leave the uniform state, as shown in Fig. 3b. Next, Fig. 3c,d show that the critical nodes denoted by the green and blue stars rapidly differentiate from the uniform state. Finally, triggered by these growing perturbations, other nodes leave the steady state to establish a non-uniform pattern (Fig. 3e, see also Supplementary Movie S1).

**Figure 4:**
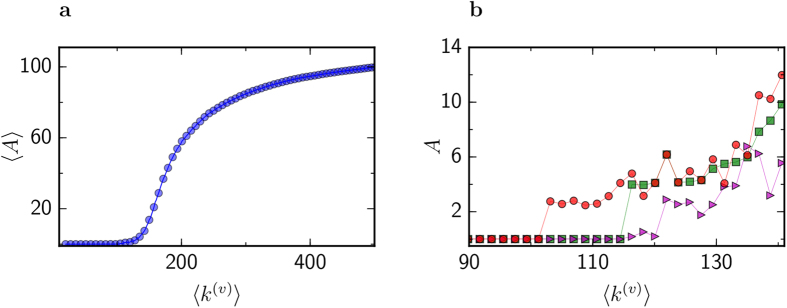
Amplitude of non-uniform patterns. (**a)**, Average amplitude of non-uniform pattern is shown as a function of 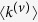 for 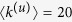 and *σ*^(*u*)^ = *σ*^(*v*)^ = 0.12. Average is taken over ten numerical simulations for different implementation of *G*^(*v*)^ with the same mean degree 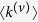. (**b),** Amplitude in the vicinity of transition for three numerical simulations where different perturbations were applied to the same sequence of networks *G*^(*v*)^.

Multistability corresponding to the cyan area of Fig. 2b has been studied via numerical simulations. Figure 4a shows the amplitude 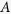 of the observed patterns (see Methods section), averaged over different simulations. Each point of the diagram is the average of ten different implementations of *G*^(*v*)^ with the same mean degree 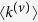; *G*^(*u*)^ is fixed. We clearly see that the amplitude is zero; *i.e.*, the uniform state is the only stable attractor of the system, for 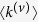 smaller than a critical threshold 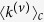. However, a more detailed look in the vicinity of this transition reveals that a number of different stationary patterns could be identified for the same parameter values. As an example, Fig. 4b shows the amplitudes in three simulations where different perturbations have been applied to the same sequence of multiplex networks. Starting from the uniform state with small perturbations, the instability occurs at some critical threshold, resulting in a small abrupt increase of amplitude. Different perturbations result in different values for the instability threshold.

**Figure 5:**
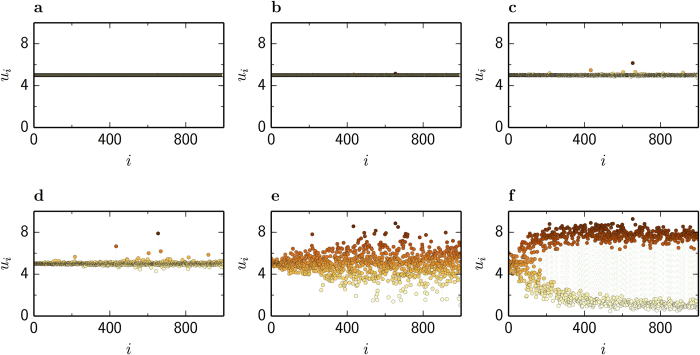
Development of non-uniform pattern. The Mimura-Murray model with mobilities *σ*^(*v*)^ = *σ*^(*u*)^ = 0.12 on a multiplex network with scale-free layers of *N* = 1,000 nodes, and mean degrees 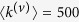 and 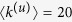. Small perturbations are added to the uniform steady state and nodes that satisfy condition (3) loose their stability and leave the uniform state. Snapshots of the activator pattern at time *t* = 11.5 (**a**), *t* = 13.5 (**b**), *t* = 15 (**c**), *t* = 16 (**d**), *t* = 18 (**e**) and *t* = 500 (**f**) are shown (see also Supplementary Movie S2). Nodes are ordered according to decreasing degrees *k*^(*u*)^.

**Figure 6:**
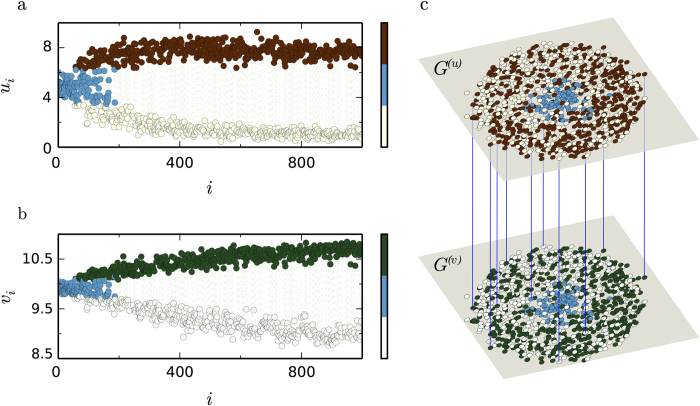
Actual multiplex pattern. The Mimura-Murray model with mobilities *σ*^(*v*)^ = *σ*^(*u*)^ = 0.12 on a multiplex network with scale-free layers of *N* = 1,000 nodes, and mean degrees 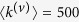 and 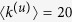. Non-uniform stationary pattern is shown in the activator layer *G*^(*u*)^ (**a**) as well as in the inhibitor layer *G*^(*v*)^ (**b**). Nodes in activator layer are ordered according to decreasing degree; nodes in the inhibitor layer are ordered correspondingly. **c**, Same pattern is shown in the actual multiplex network. Nodes in *G*^(*u*)^ are plotted using a spring algorithm, so that, those having high degrees are placed in the center and those with small degrees in the periphery. Nodes in *G*^(*v*)^ follow the same indexing. For convenience, intra-layer links are not shown, while from inter-layer links only few are chosen to be shown.

Obviously, different 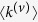 values lead to patterns of different amplitudes. Figure 3e shows a pattern for 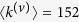, close to the transition. However, patterns where more nodes leave the uniform state can also develop far from the transition. Figure 5a–f show the evolution of small perturbations in the uniform state and the formation of a non-uniform pattern in a multiplex network with scale-free layers of *N* = 1,000 nodes, and mean degrees 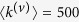 and 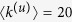. Under the influence of small perturbations, some critical nodes differentiate rapidly from the uniform steady state. Afterwards, nonlinear effects (which are not described by our theory) drive the multiplex system to self-organize into a stationary pattern with two separate group of nodes (see also Supplementary Movie S2). The separation between nodes of low and high activator densities is more pronounced in nodes with small degrees *k*^(*u*)^, while nodes with large *k*^(*u*)^ tend to sustain their initial state. Figure 6a,b show this pattern in the activator and inhibitor layers respectively, whereas Figure 6c shows the actual multiplex pattern.

## Discussion

We have proposed a new class of dynamical systems, multiplex reaction networks, where each reacting species occupies its own network layer and reacts with the other species using cross-layer contacts. As a demonstration of this new reaction scheme, we investigate pattern formation induced by diffusive transport in a multiplex network with two reacting species. Our theory, based on linear stability analysis with perturbations around the uniform steady state, correctly predicts the instability threshold observed in numerical simulations of the multiplex network.

If the different layers have the same architecture, *i.e. L*^(*u*)^ = *L*^(*v*)^, then this multiplex diffusion-induced instability reduces to the well-known Turing instability which may occur when the inhibitor diffuses much faster than the activator. Our theory (3) predicts that the analogous instability can also appear in multiplex reaction networks by increasing the inhibitor diffusion rate. However, a significantly different mechanism can trigger the formation of Turing patterns in multiplex reaction networks, even if the two species have the same mobilities (*σ*^(*u*)^ = *σ*^(*v*)^). This new instability mechanism is related to the degree combination (*k*^(*v*)^, *k*^(*u*)^) of a pair of nodes. The basic condition for any given pair of nodes *i*^(*v*)^, *i*^(*u*)^ to undergo instability is that their degrees *k*^(*v*)^ and *k*^(*u*)^ must satisfy equation (3). Indeed, this Turing-like instability always takes place for any *k*^(*u*)^ which is less or equal to the value calculated from equation (3), for a given large *k*^(*v*)^.

Similar to simplex networks, multiplex systems exhibit multistability. The onset of pattern formation can occur even before the instability described by equation (3). The minimal condition for developing non-uniform Turing patterns is that in a pair of nodes *i*^(*v*)^, *i*^(*u*)^ the degree *k*^(*u*)^ is less than or equal to the value on the saddle-node bifurcation curve that corresponds to *k*^(*v*)^. In the multistability regime, different stationary patterns can coexist with the uniform steady state for the same parameter values. However, multistability can be eliminated if the degrees of nodes in both layers are very large, so that the saddle-node and the transcritical bifurcation merge together (see Fig. 2b).

Although the observed properties of the stationary patterns are similar to those found in simplex networks^26^,^27^, the cause of destabilization of the uniform steady state is different. This cause is only characteristic of multiplex networks and lies in the relationship between *k*^(*v*)^ and *k*^(*u*)^ for a pair of nodes. Therefore, the purposeful design of nonequilibrium patterns should be possible by tuning the architecture of the multiplex structure. Recently, new algorithms for building multiplex networks with positive or negative degree correlations across the layers have been proposed^43^,^44^,^45^,^46^. Using these algorithms, we can design multiplex networks where the onset of instability is controlled by tuning the degrees *k*^(*v*)^ and *k*^(*u*)^, and the source of instability can be located at any desired pair of nodes *i*^(*v*)^, *i*^(*u*)^.

Multiplex networks can be used to represent different types of interaction^35^,^37^,^41^ or different transportation lines^38^,^40^,^47^ between discrete nodes. In ecological multiplex networks, for example, pairs of nodes might represent separate habitat patches which communicate through dispersal connections. However, prey and predators may use different connections (such as forest paths, rivers and tributaries or various transportation systems) to move among the fragmented habitats. Often, predators have more choices to move; in our representation their layer is more densely connected than the prey’s layer. This is exactly the sort of situation that favors the Turing-like instability and the subsequent establishment of Turing patterns. Considering that self-organized patterns can be found in real ecosystems^8^,^9^ it is possible that such patterns can also be observed in natural ecological systems for which the multiplex structure is innate.

## Methods

### Layer architecture

In the numerical simulations, each layer is a scale-free network constructed by the preferential attachment algorithm^48^. The network structure is determined by a symmetric adjacency matrix 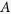, whose elements A_ij_ are 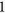 if there is a link connecting nodes *i* and *j*, and 0 otherwise. The degree, *i.e.* number of links, of node 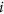 is defined as 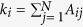. The network Laplacian matrix *L* is given by the expression 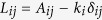.

The activator’s network *G*^(*u*)^ was constructed with mean degree 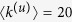. The same network was used throughout all numerical simulations. Each simulation uses a different realization of the inhibitor’s network *G*^(*v*)^, whose mean degree 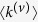 is varied between simulations. The superscripts (*u*) and (*v*) refer to activator and inhibitor. For convenience, the indices *i*^(*u*)^ of nodes in the layer *G*^(*u*)^ are assigned in order of decreasing degrees 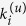: that is, 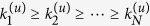. The nodes *i*^(*v*)^ in the layer *G*^(*v*)^ follow the ordering of their counterpart in *G*^(*u*)^, so for example node 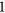 in the inhibitor network (the most highly connected node) always corresponds to node 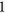 in the activator network, but the latter may or may not be highly connected.

### Multiplex networks

The multiplex networks used in our numerical simulations consist of two separate layers and two different types of links, *intra-layer* and *inter-layer* links. *Intra-layer* links are described by the adjacency matrices and limit the diffusional mobility of the species. *Inter-layer* links connect every node *i*^(*u*)^ of layer *G*^(*u*)^ to its counterpart *i*^(*v*)^ in layer *G*^(*v*)^. They represent the reaction dynamics defined in the functions *f*(*u*_*i*_*,v*_*i*_) and *g*(*u*_*i*_*,v*_*i*_).

### Activator-inhibitor dynamics

We choose the Mimura-Murray model^42^ as an example of an activator-inhibitor system. In this model the dynamics are given by the functions 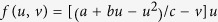 and *g*(*u,v*) = (*u *− *dv *− 1)*v*, where *u,v* correspond to the densities of activator and inhibitor respectively. The chosen parameters are *a* = 35, *b* = 16, *c* = 9, *d* = 0.4, yielding the linearly stable fixed point (*u*_0_, *v*_0_) = (5,10). This requires the networks to satisfy tr(*J*_(*u*0, *v*0)_)<0 and det (*J*_(*u*0,*v*0)_)>0, where *J* is the Jacobian matrix 
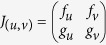
, and 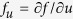, 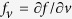, 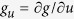 and 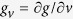 are partial derivatives.

### Linear stability analysis

The linear stability analysis is performed using a perturbation method. We introduce small perturbations 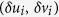 to the uniform steady state (*u*_0_*,v*_0_), as 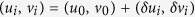. Substituting into equations (1, 2), we obtain the linearized differential equations 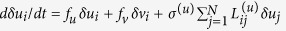 and 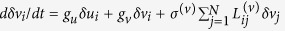. Alternatively, the linearized differential equations can be written as 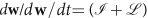 w, where 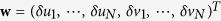 is the perturbation vector, 
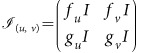
 and 
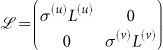
; 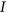 is the N×*N* identity matrix. For the linear stability analysis, the perturbation vector 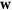 should be expanded over the set of eigenvectors of the matrix 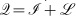. It is, however, difficult to calculate them for different network topologies, *i.e.* different Laplacian matrices 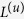 and 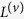. Here we propose an approximation technique to analyze the linear stability of the system. Matrix 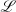 is split into 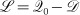, where 
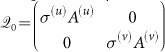
 and 
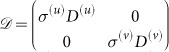
. The matrices *A*^(*u*)^ and *A*^(*v*)^ are the adjacency matrices of layers *G*^(*u*)^ and *G*^(*v*)^, respectively. The matrices *D*^(*u*)^ and *D*^(*v*)^ are the corresponding degree matrices, which have the nodes degrees in the main diagonal and are zero elsewhere. Then, matrix 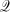 can be rewritten as 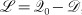, where 
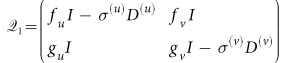
. Examining matrices 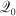 and 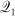, the first has elements with values of order 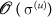 or 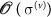, while the second has elements with values of order 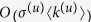 or 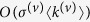. If both layers are dense enough that 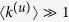 and 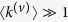, we can clearly see that the elements of matrix 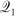 have larger values than those of matrix 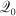, so that 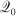 can be neglected. This approximation yields the approximate linearized equation 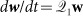. The characteristic equation for the eigenvalues *λ* is then given by 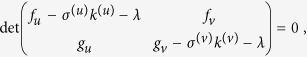 and is the same for each pair of nodes *i*^(*v*)^, *i*^(*u*)^.

This approximation neglects entirely the matrix *Q*_0_, which is associated with the precise architectures of the layers. Instead, each node is characterized only by its degree. This is quite similar to the powerful mean-field methods used for analyzing Turing patterns in single-layer networks^26^,^27^, and is always valid for multiplex networks consisting of layers with large mean degrees.

### Amplitude of non-uniform patterns

The amplitude of a non-uniform pattern is quantified as 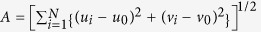.

## Additional Information

**How to cite this article**: Kouvaris, N. E. *et al.* Pattern formation in multiplex networks. *Sci. Rep.* **5**, 10840; doi: 10.1038/srep10840 (2015).

## Supplementary Material

Supplementary Information

Supplementary Movie 1

Supplementary Movie 2
