# Supplementary material for: Pattern formation in multiplex networks
Source: Sci Rep. 2015 Jun 4;5:10840. doi: 10.1038/srep10840 (PMC4455352; doi:10.1038/srep10840)
Supplement: Supplementary Information [file srep10840-s1.pdf]

Pattern formation in multiplex networks -Supplementary information-

Nikos E. Kouvaris[1,*], Shigefumi Hata[2], and Albert Díaz-Guilera[1]

[1]Department of Physics, University of Barcelona, Martí i Franqués 1, E-08028, Barcelona, Spain
[2]Department of Mathematical Science and Advanced Technology, Japan Agency for Marine-Earth Science and Technology, 236-0001 Kanagawa, Japan
[*]nikos.kouvaris@ub.edu

ABSTRACT

Supplementary movies showing the formation of Turing patterns in multiplex networks are available.

Supplementary Movie S1 shows the formation of Turing pattern for the Mimura-Murray model with $\sigma^{(v)} = \sigma^{(u)} = 0.12$ on a multiplex network with scale-free layers of $N = 1,000$ nodes and mean degrees $\langle k^{(v)} \rangle = 152$ and $\langle k^{(u)} \rangle = 20$. Nodes are ordered according to decreasing degrees $k^{(u)}$. This movie demonstrates in time the pattern formation mechanism that is described in Fig. 3 (b-e). Nodes denoted by stars are the same nodes that are denoted by stars in Fig. 3.

Supplementary Movie S2 shows the formation of Turing pattern for the Mimura-Murray model with $\sigma^{(v)} = \sigma^{(u)} = 0.12$ on a multiplex network with scale-free layers of $N = 1,000$ nodes and mean degrees $\langle k^{(v)} \rangle = 500$ and $\langle k^{(u)} \rangle = 20$. Nodes are ordered according to decreasing degrees $k^{(u)}$. This movie demonstrates in time the pattern formation mechanism that is described in Fig. 5 (a-f).
